# Predicting Site Energy Usage Intensity Using Machine Learning Models

**DOI:** 10.3390/s23010082

**Published:** 2022-12-22

**Authors:** Soualihou Ngnamsie Njimbouom, Kwonwoo Lee, Hyun Lee, Jeongdong Kim

**Affiliations:** 1 Department of Computer Science and Electronic Engineering, Sun Moon University, Asan 31460, Republic of Korea; 2 Division of Computer Science and Engineering, Sun Moon University, Asan 31460, Republic of Korea; 3 Genome-Based BioIT Convergence Institute, Sun Moon University, Asan 31460, Republic of Korea

**Keywords:** sensor network, energy usage, artificial intelligence, machine learning

## Abstract

Climate change is a shift in nature yet a devastating phenomenon, mainly caused by human activities, sometimes with the intent to generate usable energy required in humankind’s daily life. Addressing this alarming issue requires an urge for energy consumption evaluation. Predicting energy consumption is essential for determining what factors affect a site’s energy usage and in turn, making actionable suggestions to reduce wasteful energy consumption. Recently, a rising number of researchers have applied machine learning in various fields, such as wind turbine performance prediction, energy consumption prediction, thermal behavior analysis, and more. In this research study, using data publicly made available by the Women in Data Science (WiDS) Datathon 2022 (contains data on building characteristics and information collected by sensors), after appropriate data preparation, we experimented four main machine learning methods (random forest (RF), gradient boost decision tree (GBDT), support vector regressor (SVR), and decision tree for regression (DT)). The most performant model was selected using evaluation metrics: root mean square error (RMSE) and mean absolute error (MAE). The reported results proved the robustness of the proposed concept in capturing the insight and hidden patterns in the dataset, and effectively predicting the energy usage of buildings.

## 1. Introduction

The term “climate change” refers to the long-term shift in weather patterns linked to the continuous increase in atmospheric greenhouse gases [1] ($CO_{2}$, water vapor, nitrous oxide, and methane). This problem must be addressed given its negative impact on human and animal food, biomass, and crop production [2,3,4]. The combustion of fossil fuels (oil, gas, and coal) by industries to meet people’s everyday energy needs contributes to environmental shifts. With the hope of mitigating climate change, researchers have conducted many studies using ground, air, and space observation [5] and computational models [6].

According to a World Economic Forum (WEF) analysis from February 2021, the energy requirements of buildings account for 33% of greenhouse gas emissions and 40% of global energy consumption [7]. The building sector has the potential to reduce its carbon footprint via the adoption of more energy-efficient practices and the installation of cutting-edge mechanical and electrical equipment [8]. Intelligent energy management in the building sector is often accomplished via sensor devices that gather constant quantities of information on the site’s activities, allowing data-driven energy usage analysis and consequently, energy consumption control.

Previous studies have shown the usability of sensor devices in the building sectors, among which [9,10] Md. Motaharul Islam et al. presented a novel approach to structural health monitoring by sensor payload compression using arithmetic algorithms. The proposed model succeeded in a lossless data transfer paradigm and was comparable to the state-of-the-art architecture using the Huffman algorithm. M. Frei et al. [11] proposed an easy-to-implement open-source wireless sensor network (WSN) to help researchers in all fields collect data for building performance estimation. Sensor networks could therefore collect large-scale, continuous, and real-time data over time [12].

Throughout the years, with the ever-increasing amounts of data, computational models (machine learning-based solutions) have proven effective in addressing a diverse range of practical issues in public healthcare [13,14,15], bioinformatics [16], natural language processing (NLP) [17], and many others. 

The advent of computational models combined with the abundance of data have led to the development of effective control mechanisms to match energy supply to demand a primary goal of several scientists [18], specifically systems aimed towards energy usage optimization [19].

With the goal of optimizing energy usage, advances in machine learning (ML) algorithms have allowed not only the ability to perform a large set of experiments, but also to obtain high-performant models. Several researchers have studied energy consumption prediction in various areas. The researchers in [20] developed a cross-layer solution for energy optimization that could reduce energy consumption needed by 25% in printing operations [21]. Wang et al. compared various ML (support vector regression–SVR, radial basis functional neural network, general regression neural network, and propagation neural network) models for predicting hourly energy consumption in residential areas, with SVR outperforming all the other models. Likewise, the authors of [22] compared artificial neural networks (ANN), the grey model, and regression models for annual energy consumption in urban residential buildings located in urban areas. Chen Fan et al. [23] investigated the advantages of recurrent neural network (RNN)-based strategies. They designed various prediction strategies using a direct, recursive, multi-input, and multi-output approach for short-term building energy prediction. The authors of [24,25] researched how to reduce energy consumption in buildings’ heating, ventilation, and air conditioning (HVAC) systems. Ahmad MWMM et al. [24] compared ANN to random forest (RF) in terms of the standard evaluation metrics (MAPE, RMSE, mean absolute deviation (MAD), coefficient of variation (CV), mean absolute percent deviation (MAPD)) for regression tasks and concluded that regardless of the high performance reported by the RF, ANN was superior at predicting hourly HVAC electricity usage. Similarly, [25] applied the four ML (Gaussian process regression model, ANN, Gaussian mixture regression model (GMR), and change-point regression model) methods and compared their performance results. Thus, they concluded that the GMR outperformed the other algorithms due to its low RMSE rate. The ANN was proven to capture non-linear relationships among data attributes during their experimentations, but performed the worse among the ML methods employed.

The current study’s goals are: to model an energy utility prediction procedure utilizing ML algorithms based on building characteristics and weather data collected via sensors; to highlight the importance of data preprocessing in prediction tasks; and to provide a prediction module that could be used to construct an assistive application to ease site management in energy saving.

The remainder of this research work is organized as follows: Section 2 presents the proposed procedure for predicting site energy utilization, including data generation, preprocessing, and ML methods overviews; Section 3 describes the experimentation in which we presented the workstation used to conduct our experiments, described the dataset used, mentioned the hyperparameters setup, and presented the results; Section 4 discusses the paper and mentions the future work direction; and finally, Section 5 concludes the paper.

## 2. Proposed Procedure

**Figure 1:**
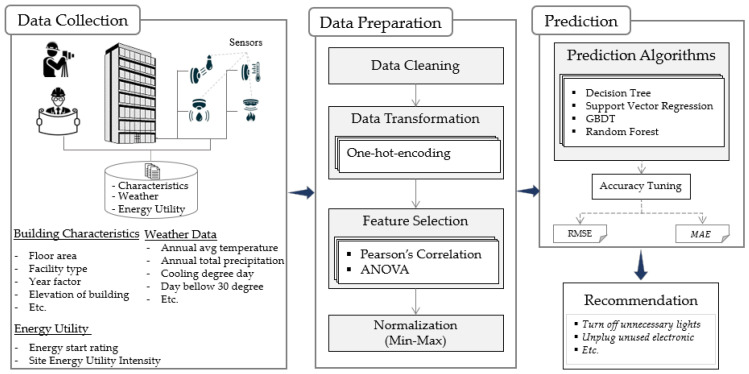
Conceptual representation of the proposed procedure.

This study aims to show the application of ML in sensor-related fields, specifically in predicting site energy utilization from sensor-collected data and other parameters as described in the dataset section. Figure 1 is an illustration of the overall conceptual model. The proposed procedure can roughly be subdivided into three stages. The first is the data collection stage. The collected data is then preprocessed to clean and remove unnecessary features, and then the data is processed to produce appropriate features for the next stage. Finally, the prediction stage involves investigating various ML algorithms and tuning their hyperparameters to obtain the performant models.

### 2.1. Dataset Collection

The dataset used in this study was obtained from Kaggle, which was made available by the Women in Data Science (WiDS) Datathon 2022 [26]. The WiDS provided data that can be divided into three main groups: first, the building’s characteristics (consisting of features such as floor area, facility type, year of building’s construction, and more; see “supplementary material Table S1”); second, the on-site sensors gathered meteorological data from the various buildings’ locations (including yearly average temperature, annual total precipitation, energy start rating, and more; see “supplementary material Table S1”); and finally, the building energy utility (including energy start rating and site energy use) was gathered over seven years. We applied a set of ML algorithms to predict the energy consumption of diverse buildings. Overall, we utilized 75,757 samples: 60,606 for training and validation, and 15,151 for testing.

### 2.2. Preprocessing

**Figure 2:**
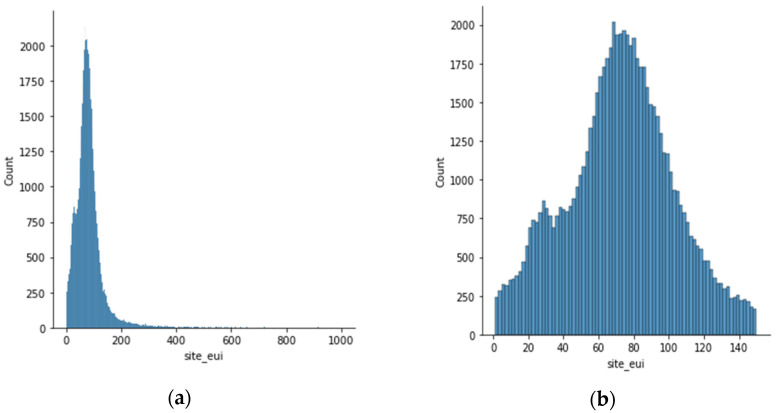
Graphical representation of outlier removal in the dataset. (**a**) Before skewness removal. (**b**) After skewness removal.

In this stage, we cleaned our data and only kept the necessary features deemed important for our prediction stage. The first operation performed was to remove the outliers due to the high skewness level in relation to the output label feature (site_eui), as shown in Figure 2. Following that, we addressed the issue of empty cells by removing features with 50% or more of empty cells. On the other hand, features with fewer miss values were filled with the most occurring value column-wise. Then, we encoded the categorical feature into the numerical data necessary for the proper execution of the models. This encoding process was performed using a one-hot encoding technique [27,28]. Moreover, we applied two feature selection techniques: the Pearson correlation and the analysis of variance. The Pearson correlation method selects features based on coefficient values between −1 and 1. Because of their linear dependence, highly correlated features have the same effect on the target value. As a result, if two features are highly correlated, one must be dropped. When determining if there is a statistically significant variation across features in a dataset, the F-test is used by the ANOVA feature selection method. The obtained value can be used to define the impact of one or more independent features on the target value (in our case, “site energy utility”). The more equal the variance between a set of features, the less weight the feature has on predicting the outcome. As a result, less critical features are discarded while the important ones are retained in our data to avoid the consequence of dimensionality and select the most valuable set of attributes to build the best models. Finally, we performed normalization on the data; this is a trivial step since it allowed us to generalize all the attributes and rescale their values between a range of 0 to 1 [29], making those values closer to one another and allowing the ML algorithm to learn quickly. In our case, we employed the min-max algorithm, which converts the lowest feature value to 0 and the highest value to 1 while preserving the relationships among the original data values. Equation (1) shows how a new normalized value is obtained using the min-max.

(1) $$X_{norm}=\frac{X-\min\left(x\right)}{\max\left(x\right)-\min\left(x\right)}$$

where $X_{norm}$ represents the normalized values (converted into data points in the range of 0 to 1); min(x), the min value in the input feature, X; max(x), the maximum value of the input attribute, X; and X, the input attribute to be normalized.

### 2.3. Overview of the Used Machine Learning Algorithms 

This section reviews the different ML algorithms used to predict the building’s energy usage using WiDS-provided data. Among the ML algorithms used, we can enumerate the random forest, decision tree, support vector regressor, and gradient boost decision tree.

- Random forest (RF): is a versatile ML algorithm that can be used for both regression and classification problems [30]. It is an ensemble ML algorithm consisting of multiple decision trees, adding more randomness as the forest grows. When compared to other ML methods, RF has additional advantages such as providing an estimation of the input variables’ importance, its lower sensitivity to noise compared, handling missing values, avoiding overfitting, etc., allowing it to achieve higher performances. Because our research was conducted on a dataset with continuous output labels, we used regression [31] rather than classification. The RF operates by constructing a collection of DTs from various combinations of samples and taking the average results obtained by those trees.
- Gradient boost decision tree (GBDT) regressor: is an ensemble learning technique for regression problems that consists of weak DT learners to produce the final output. GBDT algorithms considerably minimize the loss function and optimize the predictions by implementing a parallel learning approach via a gradient boost. GBDT also prevents overfitting and low learning time [32].
- Decision tree (DT) regressor: is a decision support mechanism with a tree-like structure representing the input features as nodes with test outcomes represented by branches. Using the dataset attributes and following the entropy concept, the DT is built in a top-down fashion following the recursive partitioning methodology, called CART [33], The root node represents the most critical predictor. The DT node homogeneity, branches’ construction, and leaf node values are obtained from Equations (2) and (3), respectively.

(2) $$SD=\sqrt{\frac{\sum_{i}^{n}{\left(x_{i}-{\bar{x}}_{i}\right)}^{2}}{n}}$$

(3) $$CV=\frac{SD}{\bar{x}}\times 100\%$$

$$\bar{x}=\frac{\sum_{i}^{n}x_{i}}{n}$$

- Support vector regressor (SVR): is a parametric regression algorithm that uses a kernel function to manipulate and fit the data samples so that in a high-dimension space, a non-linear decision surface can be transformed into a linear one described by Equation (4). SVR’s objective is to find the optimal hyperplane that minimizes the absolute error, $L_{\varepsilon }$, to that of the maximum allowed threshold error range (named epsilon) as shown in Equation (5), where $\|w{\|}^{2}$ is the Euclidean norm of the vector, w.

(4) g(x)=w·φ(x)+b

(5) $$min\frac{1}{2}\|w{\|}^{2}$$

$$L_{\varepsilon }\left(y,g\left(x\right)\right)=\{\begin{array}{cc} 0, & \;if\;\left|g\left(x\right)-y\right|\;\leq \varepsilon \\ \left|g\left(x\right)-y\right|-\varepsilon , & \;otherwise \end{array}$$

Some error points outside the epsilon error boundary could be allowed by introducing the slack variable, ξ. A new objective function can be obtained by adding the slack variable deviation to the maximum threshold error, as shown in Equation (6).

$$min\frac{1}{2}\|w{\|}^{2}+C\sum_{j=1}^{m}\left|\xi_{j}\right|$$

(6) $$L_{\varepsilon }\left(y,g\left(x\right)\right)=\{\begin{array}{cc} 0, & \;if\;\left|g\left(x\right)-y\right|\;\leq \varepsilon +\left|\xi_{j}\right|\\ \left|g\left(x\right)-y\right|-\left(\varepsilon +\left|\xi_{j}\right|\right), & \;otherwise \end{array}$$

where *C* is a constant used for the regularization by controlling the penalty imposed on the set of points lying outside of the epsilon error boundary.

### 2.4. Methods’ Accuracy 

Two evaluation metrics are used during this study to ensure the reliability of the predicted results; among others, we have root mean square error (RMSE) and mean absolute error (MAE).

- RMSE: is used to express the root mean squared difference between the observed actual values and the model predicted values. It is said to be used for absolute error representations.

(7) $$RMSE=\sqrt{\frac{\sum_{i=1}^{m}{\left(y_{i}-{\hat{y}}_{i}\right)}^{2}}{m}}$$

- MAE: is a simple equation to calculate the regression model evaluation metric, referred to as the average absolute error between the observations and the predictions. It is being used to evaluate the dataset residuals’ average.

(8) $$MAE=\frac{\sum_{i=1}^{m}\left|y_{i}-{\hat{y}}_{i}\right|}{m}$$

## 3. Experimentation

This section explains the experimentation performed during our study on predicting the site energy consumption. First, we describe the development environment used to run the experiments; second, we outline the data used; third, we enumerate the hyperparameters tuned to obtain the high-performant ML methods; and finally, we describe the results generated by the different ML algorithms.

### 3.1. Development Environment

The working station used to perform our experimentation, as summarized in Table 1, consists of a 64 GB (4 × 16 GB) RAM, an Intel Core i-9-9900k (3.60 Hz) processor, and a NVIDIA GPU RTX 3080 Ti x 4 with a 64 bit Ubuntu 18.04 operating system. The machine learning methods were built with CUDA version 11.2.0 on TensorFlow version 2.5.0 with python version 3.8.0.

### 3.2. Dataset

**Figure 3:**
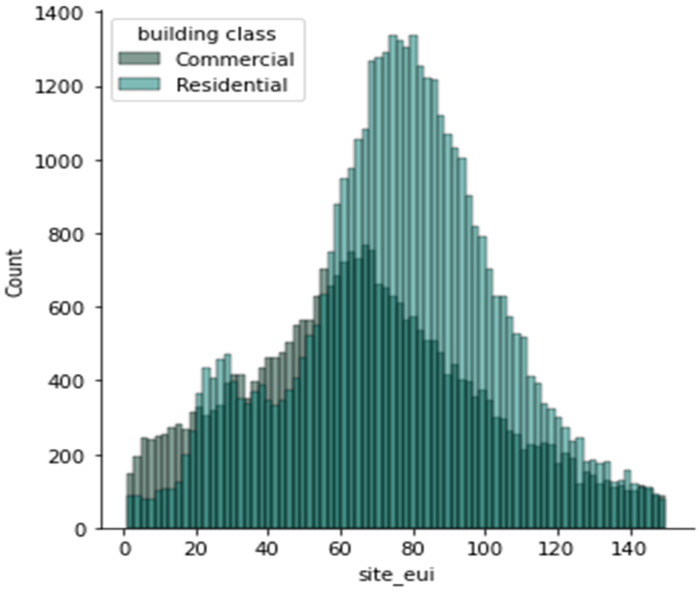
Data distribution with respect to energy utilization and building class.

The dataset provided by the Women in Data Science (WiDS) Datathon 2022 [26] was made of a 75,757 sample set of continuous, discrete, and categorical features. As described in the preprocessing, our dataset was highly skewed and had to be removed by removing the outliers, as shown in Figure 2, and then normalized using the min-max method. Figure 3 shows the data distribution for site energy consumption with respect to the building classes grouped as commercial and residential buildings. 

**Figure 4:**
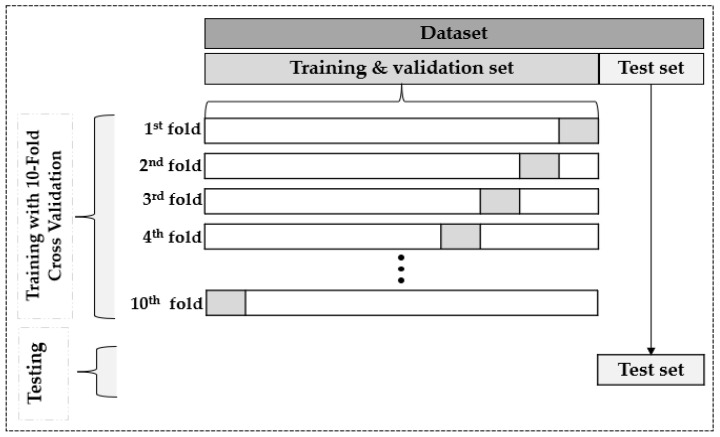
Representation of the 10-fold cross-validation and the testing performed on the dataset.

In this article, we performed a k-fold cross-validation mechanism (with k = 10) to generalize the employed ML methods. As shown in Figure 4, the sensors’ collected data from the building were shuffled and then split into the training and the test set; then, the training set was used for training and validation purposes. Throughout the k-fold cross-validation processes, the training set was subdivided into 10 subsamples. Throughout each iteration, 9 of the 10 subsamples were utilized as training datasets for model-fitting purposes while the remaining subsample served as a validation set. At the end of the 10th round, the final score was computed by averaging the scores from the previous k rounds. This mechanism has allowed us to evaluate the capability of the algorithms while trying to mitigate overfitting and obtain a less biased evaluation. As described in the previous section, the Grid_Search algorithm was performed on the different ML to find the most prevalent hyperparameters. The separated test set that the model had never seen was used to evaluate the model’s generalization performance. 

### 3.3. Hyperparameter Tuning

We used the Grid_Search method during our experiments to find the best possible combination of hyperparameters and obtain models with the lowest prediction error rates. Table 2 displays the best values obtained after GridSearch. As mentioned in the dataset section, our dataset was split in an 80:20 ratio for training and testing, respectively. The 80% training ratio was further divided and used to perform 10-fold cross-validation during which the hyperparameters were optimized.

### 3.4. Results

**Figure 5:**
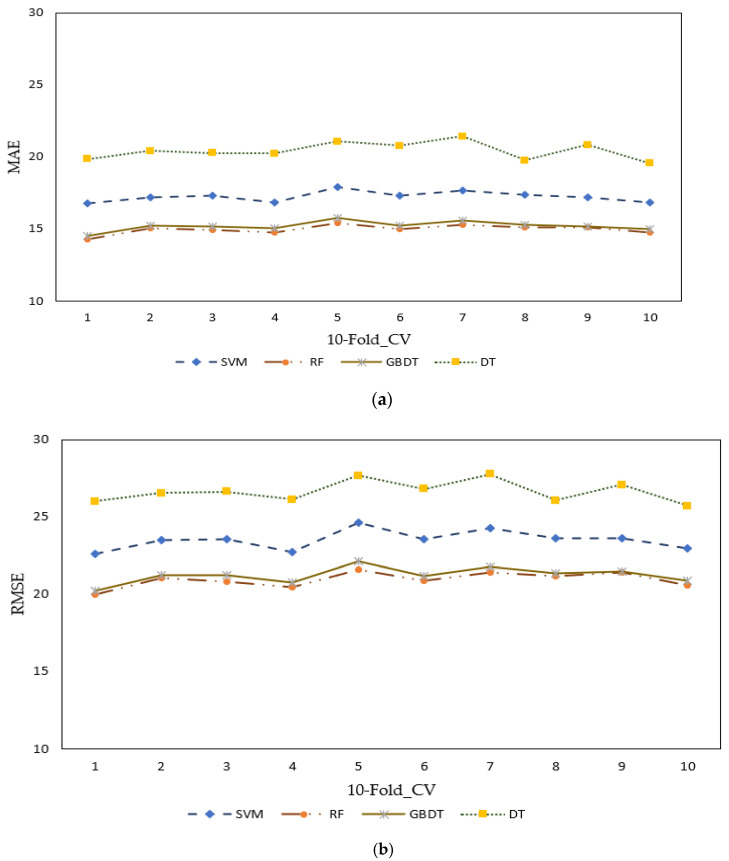
MAE and RMSE of the four ML method with 10-fold cross-validations. (**a**) Mean absolute error (MAE) graphical result representation, and (**b**) root mean squared error (RMSE) graphical result representation.

MAE and RMSE were used as indicators to illustrate the robustness of the various investigated ML methods. Figure 5 displays the k-fold (with k = 10 in our case) cross-validation results of SVM, RF, GBDT, and DT. In Figure 5a, the MAE of SVM, RF, GBDT, and DT range from 14.26 to 21.45, with RF having the lowest error rate and SVM the highest one. Similarly, when considering RMSE (Figure 5b), the various methods’ error rates ranged from 20.02 to 27.76. For both RMSE and MAE, it can be noticed that RF and GBDT have close to similar performance results.

Throughout the 10-fold operations, the error rates generated from the most optimal model (RF) were predominantly lower than the SVM and DT methods, while comparably similar to the GBDT. The utilized ML model’s statistical performance characteristics in terms of MAE and RMSE are shown in Table 3.

The results mentioned above were obtained with the help of data normalization and a proper feature selection (Pearson correlation) that allowed the models to quickly learn the hidden pattern present in the selected features while predicting the targeted energy value. Table 4 displays a feature selection-wise comparative analytical result from the 10-fold cross-validation of the four ML methods in terms of MAE and RMSE. Regardless of the feature selection used, the RF is revealed to be the most performant model. However, the models’ performances greatly varied according to the feature selection methods. These results further illustrate the importance of understanding the experimented-on data and preprocessing mechanism in ML-related prediction tasks.

Testing was performed on the last chunk of the kept-aside sample (test set) to ensure the proper generalization of our ML models and to check for overfitting. The generated error rates had satisfactory results, as listed in Table 5. Like the training stage, the RF generated the lowest MAE rate of 14.91, followed by the GBDT with 15.12. Given the nature of their architecture, these two algorithms displayed very similar performances. In contrast, both SVM and DT performed the poorest among the four ML methods, with DT yielding the highest error rate of 20.52 and 26.80 for MAE and RMSE, respectively. For each of the four methods, the resulting error rate varied between 14.91 and 20.52 for MAE and from 20.84 to 26.80 for RMSE.

## 4. Discussion

During this study, we experimented with four ML algorithms to find the most optimal method for predicting buildings’ energy utilization from data publicly made available by the Women in Data Science (WiDS) Datathon 2022 [26]. The performance of the methods was evaluated by computing their MAE and RMSE. The evaluated performance outcomes revealed that all four models could be suitable for the prediction. Nevertheless, both the RF and GBDT statistical analyses confirmed their predominance over SVM and DT with SVM performing poorly, yielding the highest error rate. The RF algorithm, characterized by its ability to effectively avoid overfitting while having a low learning time with a parallel learning pattern, achieved the lowest error rate when compared to the other algorithms. 

The results obtained in this study highlights the importance of data preprocessing, specifically outlier removal and normalization. As described in Section 3.4 (Results), the application of data normalization unveiled its utility in facilitating the model learning process [34], thereby considerably reducing the error rate of the various models, consequently leading to a model with better performance. Simultaneously, the results obtained from this study support the importance of feature selection, confirming that using proper feature selection methods could help in identifying the subset of features allowing the ML algorithms to capture distinctive aspects of the building’s characteristics [35] to effectively predict energy consumption.

For future work directions, we intend to extend our experimentation by adding more machine learning algorithms and the neural network model, using more feature selection methods, and performing an extensive comparative analysis. Furthermore, we intend to evaluate the obtained model on similar datasets; the site energy’s utilization prediction model will be implemented as an API and made publicly available based on the attained results.

## 5. Conclusions

An accurate prediction of energy usage is challenging (considering the large set of features) and is an important task to be performed. Therefore, this study attempts to model energy consumption prediction by investigating four machine learning algorithms together with data preprocessing (outlier removal, feature selection, and data normalization) and compares their performance to determine their applicability for predicting site energy consumption. The most efficient algorithm is selected based on the performance metrics: MAE and RMSE. After appropriate preprocessing of data, the RF outperformed the other methods with the following performances: 14.91 and 20.84 for MAE and RMSE, respectively. During our study, data preprocessing (outlier removal, feature selection, and data normalization) was revealed to be crucial and described in the Results section, whereby the error rate was considerably reduced and the model robustness enforced. Our model’s low error rates allowed us to make relatively accurate forecasting of the site’s energy usage, thus providing recommendations that directly minimize the consumption rate and indirectly help reduce climate change.

## Figures and Tables

**Table 1:** Environmental setup of the workstation.

Components	Description
GPU	NVIDIA RTX 3080 Ti x 4
CPU	Intel Core i-9-9900k (3.60 Hz)
RAM	64 GB (16 GB × 4)
OS	Ubuntu 18.04 64 bit
CUDA	11.2.0
TensorFlow	2.5.0
Python	3.8.0

**Table 2:** Hyperparameter tuning of the different models used.

Models	Hyperparameters	Description	Values
RF	N_estimator	# Of DT that will constitute the forest	635
	Max_feature	# Of features in each tree	auto
	Max_depth	Max depth of each DT	150
	Min_sample_leaf	# Of required samples at leaf node	1
DT	Max_depth		5
	Max_feature		Auto
	Min_samples_leaf		2
	Max_leaf_nodes	Identical to RF and GBDT	40
	Min_weight_fraction_leaf	Fraction of samples’ sum of weight required at leaf node	0.1
	Splitter	Split strategy for each node	random
GBDT	Max_depth		40
	N_estimators		142
	Max_features	Identical to parameters of RF and DT	auto
	Min_sample_leaf		63
	Subsample	Fraction of sample sets used in fitting each tree learner	0.65
	Learning_rate	Rate at which each learning tree contribute	0.05
SVM	Kernel	Type of kernel used in the algorithm	rbf
	C	Weight importance for the training data	1.0
	Gamma	Factor controlling single point distance of the influence	0.4
	epsilon	Margin of error that can be tolerated	0.2

“#” is used to refer to “Number”.

**Table 3:** Results of the various machine learning algorithms on the training set.

Models	MAE	RMSE
DT	20.61	26.87
GBDT	14.94	20.88
RF	14.63	20.54
SVM	17.25	23.52

**Table 4:** Comparative results of the four ML algorithms based on the feature selection technique used.

	Pearson Correlation		ANOVA	
Models	MAE	RMSE	MAE	RMSE
DT	20.61	26.87	21.25	27.21
GBDT	14.94	20.88	20.29	26.27
RF	14.63	20.54	21.75	28.27
SVM	17.25	23.52	23.23	29.02

**Table 5:** Results of the various machine learning algorithms on test set.

Models	MAE	RMSE
DT	20.52	26.80
GBDT	15.12	21.10
RF	14.91	20.84
SVM	17.30	23.51

## Data Availability

The data used in this study are available and can be downloaded from: WIDS Datathon 2022. https://www.kaggle.com/competitions/widsdatathon2022/data (accessed on 4 July 2022). This data are not to be used for any financial purposes.

## Supplementary Materials

The following supporting information can be downloaded at: https://www.mdpi.com/article/10.3390/s23010082/s1.
