# Supplementary material for: Predicting Site Energy Usage Intensity Using Machine Learning Models
Source: Sensors (Basel). 2022 Dec 22;23(1):82. doi: 10.3390/s23010082 (PMC9823370; doi:10.3390/s23010082)
Supplement: Supplementary file 1 [file sensors-23-00082-s001.zip › sensors-2074107_supplementary material.pdf]

Supplementary Material: Predicting Site Energy Usage Intensity using Machine Learning Models

Table S1. Description of the dataset attributes used during our experimentations.

Feature name		Description	Total values	No. missing values	Min value	Max value	Std dev	Mean
Year_Factor		Observation year of weather and energy usage (anonymous)	75757	0	x	x	x	x
State_Factor		State location of the building (anonymous)			x	x	x	x
building_class		Building classification			x	x	x	x
facility_type		Building usage type			x	x	x	x
floor_area		Building floor area (in square feet)			9.43e02	6.39e06	2.47e5	1.66e05
year_built		Construction year of building	73920	1837	1600	2015	37.05	1952
energy_star_rating		Building energy star rating	49048	26709	0.00	100.00	28.66	61.05
ELEVATION		Building elevation	75757	0	-6.40	1924.5	60.66	39.51
January	Min_temp	Building min temperature (in Fahrenheit) in January			-6.40	49	9.38	11.43
	Avg_temp	Building avg temperature (in Fahrenheit) in January			10.81	64.76	7	34.31
	Max_temp	Building max temperature (in Fahrenheit) in January			42.00	91.00	5.36	59.05
February	Min_temp	-			-13.00	48.00	12.58	11.72
	Avg_temp	-			13.25	65.11	8.87	35.53
	Max_temp	-			38.00	89.00	8.41	58.49
March	Min_temp	-			-9.00	52.00	10	21.61
	Avg_temp	-			25.85	69.76	6.57	44.47
	Max_temp	-			53.00	95.00	7.68	70.90
April	Min_temp	-			15.00	52.00	5.58	32.04
	Avg_temp	-			40.28	54.77	2.50	53.78
	Max_temp	-			62.00	104.00	4.10	82.66
May	Min_temp	-			23.00	58.00	4.20	44.71
	Avg_temp	-			46.95	82.11	2.79	63.74
	Max_temp	-			64.00	112.00	2.86	88.99
June	Min_temp	-			30.00	68.00	3.42	51.13

	Avg_temp	-			54.10	89.55	2.86	71.07
	Max_temp	-			67.00	119.00	3.60	91.40
	Min_temp	-			37.00	74.00	4.18	60.57
July	Avg_temp	-			54.82	94.44	4.09	76.60
	Max_temp	-			65.00	117.00	4.16	95.54
	Min_temp	-			31.00	77.00	4.44	58.52
August	Avg_temp	-			56.69	94.90	3.82	75.35
	Max_temp	-			66.00	116.00	4.14	92.57
	Min_temp	-			26.00	65.00	4.29	50.79
September	Avg_temp	-			53.60	90.12	3.60	69.59
	Max_temp	-			64.00	111.00	5.01	92.17
	Min_temp	-			18.00	61.00	5.09	38.21
October	Avg_temp	-			44.69	80.74	2.64	58.73
	Max_temp	-			59.00	108.00	5.58	81.14
	Min_temp	-			4.00	52.00	4	28.63
November	Avg_temp	-			4.09	67.42	4.09	48.12
	Max_temp	-			53.00	96.00	4.37	71.20
	Min_temp	Building temperature (in Fahrenheit) in December			-16.00	44.00	9.01	22.74
December	Avg_temp	Buidling avg temperature (in Fahrenheit) in December			23.79	61.79	5.87	41.63
	Max_temp	Building max temperature (in Fahrenheit) in December			42.00	86.00	6.05	64.49
cooling_degree_days		No. of degrees where the daily average Temp exceeds 65°F			0	4948.00	391.56	1202.25
heating_degree_days		No. of degrees where the daily average Temp falls under 65°F			398.00	7929.00	824.37	4324.96
precipitation_inches		Annual precipitation at building location (in inch)			0	107.69	824.37	4324.96
snowfall_inches		Annual snowfall at building location (in inch)			0	127.30	10.59	42.43
snowdepth_inches		Annual snow depth at building location			0	1292.00	175.27	167.86

avg_temp	Annual avg Temp at building location			44.51	77.25	2.24	56.18
days_below_30F	No. of days bellow 30°F at building location			0	170	27.59	48.76
days_below_20F	No. of days bellow 20°F at building location			0	93.00	14.47	17.45
days_below_10F	No. of days bellow 10°F at building location			0	59.00	7.071	4.87
days_below_0F	No. of days bellow 0°F at building location			0	31.00	2.89	0.88
days_above_80F	No. days above 80°F at building location			0	260	82.71	25.28
days_above_90F	No. days above 90°F at building location			0	185	14.059	10.944
days_above_100F	No. days above 100°F at building location			0	119	0.280	2.252
days_above_110F	No. days above 110°F at building location			0	16	0.002	0.142
direction_max_wind_speed	Wind direction for max wind speed at building location	34675	41082	1.00	360.00	66.55	131.15
direction_peak_wind_speed	Wind direction for peak wind gust speed at building location	33946	41811	1.00	360.00	62.78	130.31
max_wind_speed	Max wind speed at building location	34675	41082	1.00	360.00	4.20	6.46
days_with_fog	No. of days with fog at building location	29961	45796	1.00	360.00	109.14	50.70
site_eui	Energy usage intensity of site (utility bills)	75757	0	1.00	997.87	82.58	58.26
id	Id of the building			x	x	x	x

("X") Not applicable

("- ") Similar to the above cell
